# Downregulation of Interleukin- (IL-) 17 through Enhanced Indoleamine 2,3-Dioxygenase (IDO) Induction by Curcumin: A Potential Mechanism of Tolerance towards *Helicobacter pylori*

**DOI:** 10.1155/2018/3739593

**Published:** 2018-10-08

**Authors:** Tiziana Larussa, Serena Gervasi, Rita Liparoti, Evelina Suraci, Raffaella Marasco, Maria Imeneo, Francesco Luzza

**Affiliations:** Department of Health Sciences, University of Catanzaro “Magna Graecia”, 88100 Catanzaro, Italy

## Abstract

The anti-inflammatory and antimicrobial properties of curcumin suggest its use as an anti-*Helicobacter pylori* (*H. pylori*) agent, but mechanisms underlying its helpful activity are still not clear. Indoleamine 2,3-dioxygenase (IDO) promotes the effector T cell apoptosis by catalyzing the rate-limiting first step in tryptophan catabolism, and its high expression in *H. pylori*-infected human gastric mucosa attenuates Th1 and Th17 immune response. The aim of this study was to investigate the role of curcumin in modulating the expression of IL-17 and IDO in *H. pylori*-infected human gastric mucosa. In an organ culture chamber, gastric biopsies from 35 patients were treated with and without 200 *μ*M curcumin. In *H. pylori*-infected patients (*n* = 21), IL-17 was significantly lower, both in gastric biopsies (*p* = 0.0003) and culture supernatant (*p* = 0.0001) while IDO significantly increased (*p* < 0.00001) in curcumin-treated sample compared with untreated samples. In a subgroup of *H. pylori*-infected patients (*n* = 15), samples treated with curcumin in addition to IDO inhibitor 1-methyl-L-tryptophan (1-MT) showed a higher expression of IL-17 compared with untreated samples and curcumin-treated alone (*p* < 0.00001). Curcumin downregulates IL-17 production through the induction of IDO in *H. pylori*-infected human gastric mucosa, suggesting its role in dampening *H. pylori*-induced immune-mediated inflammatory changes.

## 1. Introduction

*Helicobacter pylori* (*H. pylori*) is a ubiquitous pathogen, and it is believed that at least 50% of the world's population has been infected [1]. Although the epidemiology of *H. pylori* infection is undergoing changes with regard to improvements in hygienic and socioeconomic conditions and most infections remain asymptomatic, there are few prospective studies in the general population, despite the infection still having a major impact on public health [2]. The colonization, if not treated, leads to a lifelong chronic gastritis, which is asymptomatic in the majority of subjects (85%). On the other hand, the involvement of the bacterium has been proven in the pathogenesis of a number of pathologies affecting the gastrointestinal tract such as peptic ulcer disease (11%) or less frequently gastric cancer and MALT lymphoma of the stomach (1%) [3]. The human host mounts an innate and adaptive immune responses against the bacterium, but this is not enough to clear the infection [4]. Indeed, *H. pylori* is able to manipulate the responses of the T helper cells and their signature cytokines, avoiding its clearance by the host immune system [5]. Th1 polarization occurring during *H. pylori* infection is well documented, but evidences suggest its modulation by the bacterium, which in this way allows the persistence of the infection and the development of *H. pylori*-related burden of diseases [6].

Interleukin- (IL-) 17A (hereafter referred to as IL-17) is the key cytokine which is produced by Th17 cells and acts as a mediator in the host inflammatory defenses, both against extracellular bacterial and pathogenic fungi [7]. During human *H. pylori* infection, a higher amount of IL-17 has been found in the gastric mucosa, which was able to enhance IL-8 production, and was accompanied by a more pronounced level of gastritis [8].

Indoleamine 2,3-dioxygenase (IDO) is a heme-containing enzyme that promotes the apoptosis of effector T cells by catalyzing the rate-limiting first step in tryptophan (Trp) catabolism via the kynurenine (Kyn) pathway [9]. On the other hand, IDO is involved in the differentiation of naïve T cells, promoting the switch towards T regulatory cells (Treg cells) [10]. Moreover, a role for IDO in the regulation of IL-17 production has been documented in several animal models of disease [11–13]. We previously demonstrated that a high amount of IDO in the human gastric mucosa infected by *H. pylori* attenuates Th1 and Th17 immune responses, suggesting an involvement of this enzyme in the mechanisms by which *H. pylori* is able to promote its pathogenicity and establish a condition of immunological tolerance [14].

Curcumin belongs to the class of phenols called curcuminoids, being the most representative one, and is isolated from the plant *Curcuma longa*, which is known for the common use as a spice, food coloring, and preservative. In a mouse model of gastritis sustained by *H. pylori*, curcumin was able to downregulate the expression of inflammatory cytokines and chemokines, as well as toll-like receptors, all figures which are used by the bacterium to establish and maintain the infection [15]. Although these anti-inflammatory and antimicrobial properties suggest a potential use of curcumin as an anti-*H. pylori* agent, mechanisms that underlie its beneficial activity are still not clear [16].

The aim of this study was to investigate the role of curcumin in modulating the expression of IL-17 and IDO in *H. pylori*-infected human gastric mucosa, thus providing novel insights into the knowledge of the immune pathways occurring during *H. pylori* infection.

## 2. Materials and Methods

### 2.1. Patients and Samples

Thirty-five patients (20 M, 15 F, median age 47.5 years, range 20–75) underwent esophagogastroduodenoscopy for their dyspeptic symptoms, and biopsy specimens were collected in the antrum, in order to perform the urease quick test (Eurospital, Trieste, Italy, 1 biopsy), histology (1 biopsy), and organ culture (2–4 biopsies). Then, patients were classified as *H. pylori*-infected (*n* = 21) or not (*n* = 14) according to the results of the urease quick test, histology, and ^13^C-urea breath test (Richen Europe, Milan, Italy); among the three tests, two positive tests were needed to consider the patient as infected, while the negative result of all the three tests confirmed as not infected. When only one among the three abovementioned tests resulted positive, patient was not included in the analysis. No previous therapy for *H. pylori* was allowed, neither treatment with antibiotics nor nonsteroidal anti-inflammatory drugs (NSAIDs) over an 8-week period before the study. Chronic inflammatory conditions (e.g., diabetes, chronic renal failure, inflammatory bowel diseases, or rheumatic diseases) were absent in all patients enrolled.

### 2.2. Gastric Biopsy Culture

After collection, biopsy specimens were immediately positioned on steel grids in the central well of an organ culture dish containing RPMI 1640, 5% fetal bovine serum, 10 mmol/L L-glutamine, 0.25 *μ*g/mL amphotericin B, 10 mg/mL vancomycin, 100 mg/mL gentamicin, and trimethoprim 5 mg/mL (all from Invitrogen, Carlsbad, CA). Subsequently, each culture dish was carefully situated in an organ culture chamber with 5% CO_2_/95% O_2_ at 37°C. Biopsy specimens were stimulated or not (untreated controls) with curcumin (Sigma, St. Louis, MO, USA) using a final concentration of 200 *μ*M. Due to its poor water solubility, curcumin was dissolved in DMSO, to obtain a 10 mM stock solution. An equal amount of DMSO was added to the medium as vehicle control and used in the untreated samples. From a subgroup of patients (*n* = 20), further biopsy specimens were collected and treated with 200 *μ*M curcumin in addition to the selective IDO inhibitor 1-methyl-L-tryptophane (1-MT) (Sigma, St. Louis, MO, USA) using a concentration of 200 *μ*M. For this purpose, 1-MT was dissolved in 1 M NaOH to obtain a concentration of 1 M 1-MT and it was subsequently diluted to a 20 mM stock solution. The pH of the solution was then adjusted to 7.5 with hydrochloric acid. The addition of 1-MT always took place *ab initio* of each experiment while the 1 M NaOH solution, without 1-MT, represented the vehicle control in the untreated samples. Cultures were stopped after 20 hours; biopsies were collected and frozen and then set to −80°C for storage.

Preliminary experiments were performed in order to optimize dose ranging and time course (data not shown). To exclude direct toxicity of curcumin, DMSO, 1-MT, and NaOH during the culture, the viability of the specimens was assessed by examination of frozen tissue sections with hematoxylin/eosin (H&E) staining, after 20 hours of culture. Biopsies were considered viable when, after H&E staining, the morphology of the tissue was intact, showing a well-defined surface cuboidal epithelium of mucous cells and deeper glands of cuboidal epithelium of mucous and endocrine cells. Biopsy morphology has been evaluated by qualitative analysis using an Olympus CK41 optical microscope (Olympus Microscopy Europa).

### 2.3. Extraction of Total Proteins

Total proteins were extracted from biopsy specimens after 20 hours of culture, by means of a lysis buffer (50 mmol/L HEPES pH 7.6, 150 mmol/L NaCl, 1% Triton X-100, 1 mmol/L Na_3_VO_4_, 10 mmol/L NaF, 30 mmol/L Na_4_P_2_O_7_, 10% glycerol, 1 mmol/L benzamidine, 1 mmol/L dithiothreitol (DTT), 10 *μ*g/mL leupeptin, and 1 mmol/L phenylmethylsulfonyl fluoride (PMSF)) (all from Sigma, St. Louis, MO, USA). Samples were incubated for 30 minutes on ice, then were centrifuged for 30 minutes at 3100*g* at 4°C. The supernatant was collected, and Bradford's method was performed to assess the protein concentration, in order to guarantee an equal loading during Western blot analysis. Aliquots were stored at −80°C.

### 2.4. Western Blotting

Levels of IL-17 and IDO were measured in total proteins extracted from cultured biopsies after a 20-hour culture period. An equal amount of proteins (80 *μ*g) was resolved by sodium dodecyl sulfate-polyacrylamide gel electrophoresis and electrophoretically transferred onto an Immobilon-P membrane (Amersham Life Sciences Inc., Buckinghamshire, UK). A 12% gel and a 10% gel were used in the detection of IL-17 and IDO, respectively. The equal loading and transfer of proteins was confirmed by Ponceau S staining. Then, the membranes were put for 1 h in a “blocking buffer” (5% nonfat dry milk in 10 mmol/L Tris-HCl, 100 mmol/L NaCl, and 0.1% Tween 20, pH 7.6) and subsequently incubated with IL-17 and IDO rabbit anti-human mAbs, used at 1 : 500 dilution, overnight at 4°C. After, the membranes were incubated with horseradish peroxide-conjugated goat anti-rabbit IgG mAb, at a dilution of 1 : 2000 for 1 h. The membranes were stripped and reprobed with an anti-*β*-actin antibody at a dilution of 1 : 5000 (Sigma) and then incubated with a horseradish peroxide-conjugated goat anti-mouse IgG mAb at a dilution of 1 : 2000. Detection was allowed by means of a chemiluminescence luminol reagent. Measurement of the bands was performed densitometrically, and the relative density was calculated using the density of the *β*-actin bands in each sample. Values were expressed as arbitrary densitometric units (a.u.) in agreement with signal intensity. All the reagents were from Santa Cruz Biotechnology, Santa Cruz, CA, USA, unless otherwise specified.

### 2.5. Measurement of IL-17 in the Culture Supernatants

After 20 hours, the supernatant from each culture was collected and stored at −80°C. Measurement of IL-17 levels was performed by enzyme-linked immunosorbent assay (ELISA, R&D Systems, Minneapolis, MN, USA) following the manufacturer's instructions. The amount of cytokine was quantified within each supernatant in duplicate. Results were expressed as pg/mL; normalization was made on protein content, and the mean value with standard deviation was calculated.

### 2.6. Histology

Biopsies were fixed with 4% paraformaldehyde, and after a process of dehydration and paraffin embedding, they were cut into thick sections (3–10 *μ*m), for staining with H&E and May-Grünwald Giemsa solution. Results were blindly checked by the pathologist for *H. pylori* infection.

### 2.7. Statistics

Statistical analysis was performed using the Mann-Whitney *U* test and one-way analysis of variance (ANOVA) with the Tukey multiple comparison test, as appropriate. *p* values of 0.05 or less were regarded as statistically significant.

### 2.8. Ethical Considerations

The study protocol, approved by the local Research Ethics Committee (no. 2013.31), was in agreement with the requirements of the Declaration of Helsinki. All patients gave their written informed consent to the study procedures.

## 3. Results

### 3.1. Curcumin Decreases IL-17 in Both Mucosa and Supernatant of Gastric Biopsy Cultures

**Figure 1:**
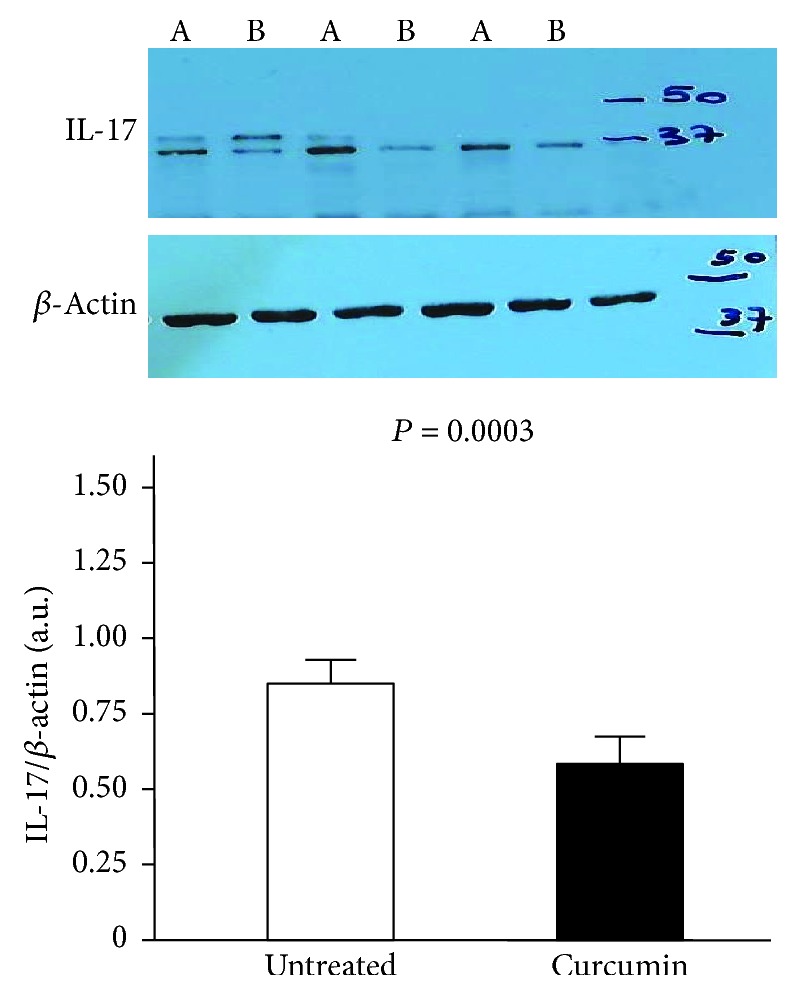
Levels of IL-17 by Western blotting in total protein extracts from gastric biopsies of *H. pylori*-infected patients (*n* = 21) treated with medium (untreated samples) or 200 *μ*M curcumin for 20 hours. The loading control was represented by *β*-actin. Values are expressed as mean values ± SD of arbitrary units (a.u.). A Western blot panel of one representative experiment is shown (A is the untreated sample and B is the curcumin-treated sample, for each patient). Statistical analysis was performed using the Mann-Whitney *U* test. *p* values of 0.05 or less were regarded as statistically significant.

**Figure 2:**
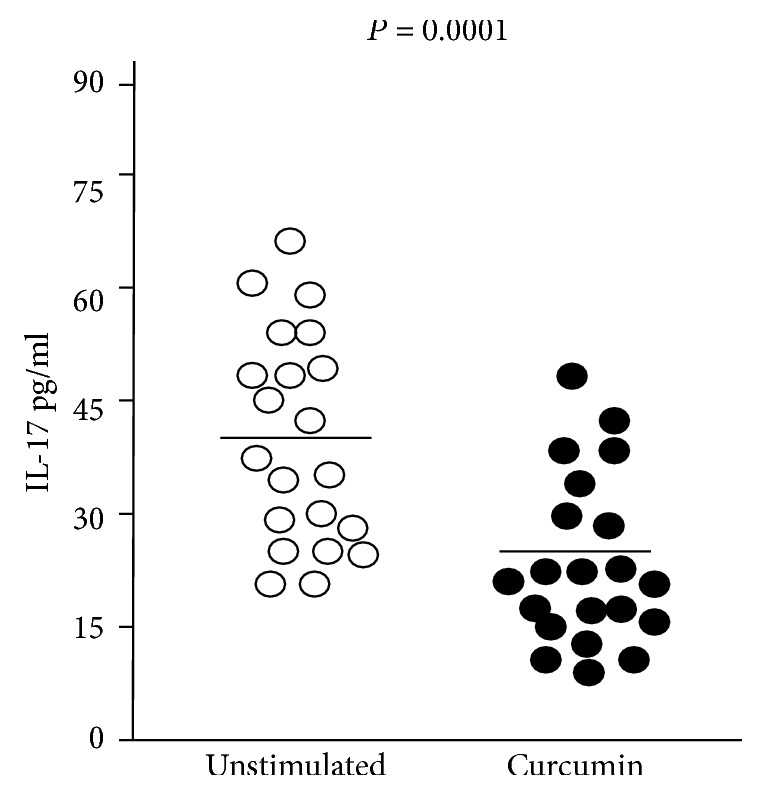
Levels of IL-17 by ELISA in supernatant from gastric biopsies of *H. pylori*-infected patients (*n* = 21) treated with medium (untreated samples) or 200 *μ*M curcumin for 20 hours. Values are given in pg/mL as scattered plots with mean values. Statistical analysis was performed using the Mann-Whitney *U* test. *p* values of 0.05 or less were regarded as statistically significant.

To evaluate the ability of curcumin to dampen the mucosal inflammation in human gastric mucosa, we measured the expression of the proinflammatory cytokine IL-17 in gastric tissue and its release in the supernatant obtained from cultured biopsy specimens. Levels of IL-17 resulted significantly lower in curcumin-treated sample compared with untreated samples, both in gastric biopsies (0.56 ± 0.15 arbitrary units (a.u.) vs. 0.80 ± 0.19 a.u., *p* = 0.0003, Figure 1) and culture supernatant (25.18 ± 10.77 pg/mL vs. 40.66 ± 11.69 pg/mL, *p* = 0.0001, Figure 2) from *H. pylori*-infected patients (*n* = 21). Levels of IL-17 obtained in the untreated samples from *H. pylori*-uninfected patients (*n* = 14) did not show significant changes after treatment with curcumin, both in gastric biopsies (0.38 ± 0.23 a.u. vs. 0.41 ± 0.21 a.u., *p* = 0.4) and culture supernatant (17.29 ± 12.92 pg/mL vs. 17.09 ± 10.88 pg/mL, *p* = 0.9).

### 3.2. Expression of IDO Is Enhanced in *H. pylori*-Infected Biopsies Treated with Curcumin

**Figure 3:**
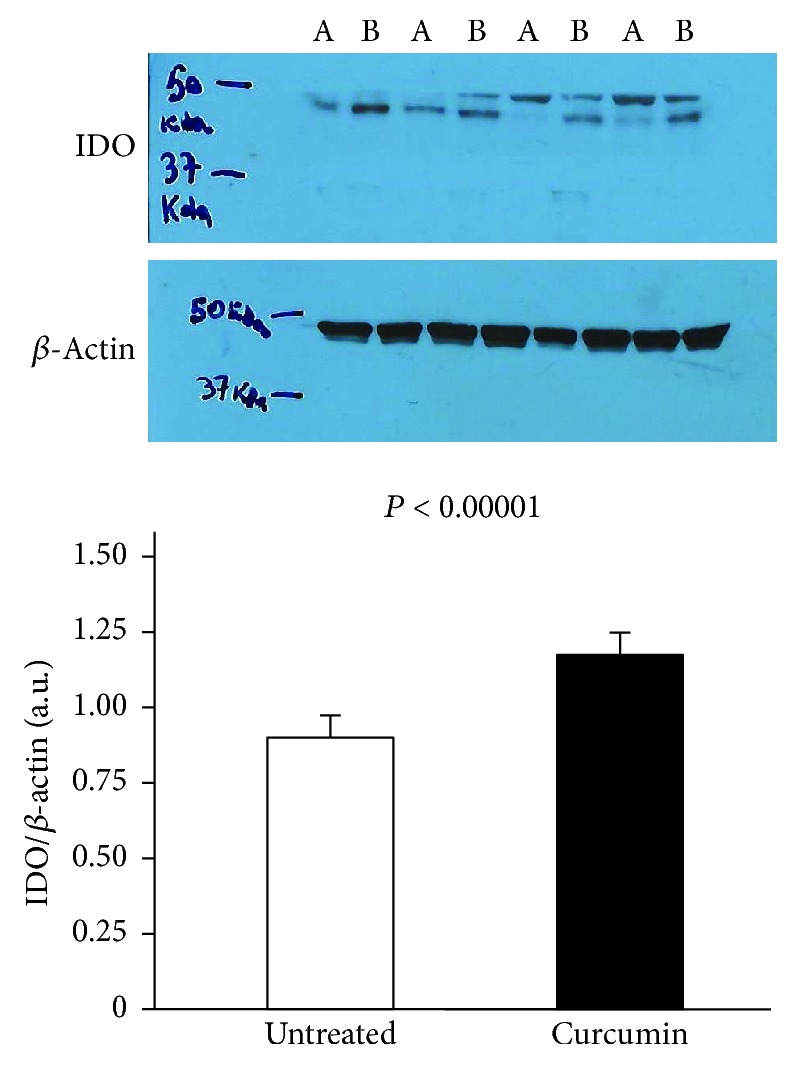
Expression of indoleamine 2,3-dioxygenase (IDO) by Western blotting in total protein extracts from gastric biopsies of *H. pylori*-infected patients (*n* = 21) treated with medium (untreated samples) or 200 *μ*M curcumin for 20 hours. The loading control was represented by *β*-actin. Values are expressed as mean values ± SD of arbitrary units (a.u.). A Western blot panel of one representative experiment is shown (A is the untreated sample and B is the curcumin-treated sample, for each patient). Statistical analysis was performed using the Mann-Whitney *U* test. *p* values of 0.05 or less were regarded as statistically significant.

In gastric biopsy cultures from *H. pylori*-infected patients (*n* = 21), IDO significantly increased in curcumin-treated sample compared with untreated samples (1.24 ± 0.15 a.u. vs. 0.89 ± 0.13, *p* < 0.00001, Figure 3). IDO expression was very low in *H. pylori*-uninfected samples (*n* = 14) and did not significantly change after treatment with curcumin.

### 3.3. IDO Inhibition Suppresses IL-17 Downregulation Induced by Curcumin

**Figure 4:**
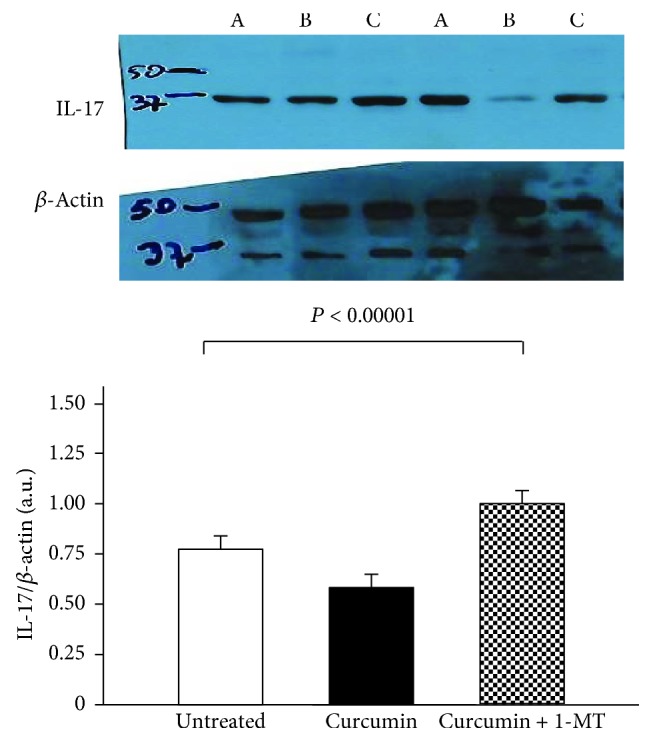
Levels of IL-17 by Western blotting in total protein extracts from gastric biopsies of the subgroup of *H. pylori*-infected patients (*n* = 15) treated with medium (untreated samples) or 200 *μ*M curcumin or 200 *μ*M curcumin plus the indoleamine 2,3-dioxygenase (IDO) inhibitor, 1-methyl-L-tryptophane (1-MT), at a concentration of 200 *μ*M for 20 hours. The loading control was represented by *β*-actin. Values are expressed as mean values ± SD of arbitrary units (a.u.). A Western blot panel of one representative experiment is shown (A is the untreated sample, B is the curcumin-treated sample, and C is the curcumin plus 1-MT-treated sample, for each patient). Statistical analysis was performed using one-way analysis of variance (ANOVA) with the Tukey multiple comparison test. *p* values of 0.05 or less were regarded as statistically significant.

In order to assess whether the activity of curcumin was affected by IDO expression, further gastric biopsies, collected from a subgroup of *H. pylori*-infected and uninfected patients, were treated both with curcumin and in the presence or absence of the IDO inhibitor, 1-MT. We evaluated the expression of IL-17 by means of Western blotting. In the subgroup of *H. pylori*-infected patients (*n* = 15), samples cultured with curcumin, in addition to IDO inhibitor 1-MT, showed a significantly higher IL-17 expression compared with untreated samples as well as with those treated with curcumin alone (1.01 ± 0.13 a.u. vs. 0.76 ± 0.17 a.u. and vs. 0.53 ± 0.13 a.u., *p* < 0.00001, Figure 4). In the subgroup of *H. pylori*-uninfected samples (*n* = 5), IL-17 levels did not significantly change after treatment with 1-MT compared with untreated samples or with curcumin treatment alone (0.28 ± 0.08 a.u. vs. 0.27 ± 0.07 a.u. and vs. 0.26 ± 0.04 a.u., *p* = 0.67).

## 4. Discussion

In this study, the data show that curcumin performs an immune-mediated anti-inflammatory activity in human gastric mucosa colonized by *H. pylori*.

At present, nutraceutical substances, including phytochemicals, bioactive peptides, and omega-3 fatty acids, are investigated for their beneficial properties in several inflammatory diseases [17, 18]. Curcumin is one of the main constituents of turmeric spice, which has been used for centuries due to its recognized benefits on human health and its lack of harmful effects. In particular, it has been recommended as an anti-inflammatory agent, although its clear mechanism of action is largely unknown [19]. Some *in vitro* reports highlighted the antibacterial activity of curcumin against *H. pylori*, as this compound was strongly effective in the inhibition of the bacterial growth and thereby providing a basis for new treatment strategies in the field of anti-*H. pylori* therapies [20]. However, there were conflicting results from human trials, suggesting that further studies are needed to clarify the activity of curcumin on humans [21]. With this in mind, gastric biopsies collected from patients with and without *H. pylori* infection have been challenged with curcumin in an organ culture system, in order to evaluate its effects in human gastric mucosa. In this model, the whole inflammatory process is more intricate than that acting in *in vitro* experiments and represents the conditions which seem to resemble more closely to those that occur in an *in vivo* setting.

The recent characterization of the Th17 cells and their signature cytokine IL-17 provided a novel communication network between the adaptive and innate immunities [22]. For this reason, we first focused our attention on IL-17, which is enhanced in the gastric mucosa during *H. pylori* infection, rules out a proinflammatory activity, and is associated with disease severity [23]. Both in the protein extracts and the supernatants of the curcumin-treated gastric biopsies from *H. pylori*-infected patients, a significant reduction in IL-17 levels was found. These results are in agreement with the documented ability of curcumin to impair development and differentiation of Th17 cells in several inflammatory diseases and, in particular, in the gastrointestinal tract [24, 25]. The mechanisms by which curcumin mediates its anti-inflammatory and antioxidant properties include the inhibition of the nuclear factor kappa-light-chain-enhancer of activated B cell- (NF-*κ*B-) related inflammatory pathway, with consequent downregulation of TNF-*α*, IL-12, and IL-2 [26]. Considering that IL-17 production has been involved in the activation of NF-*κ*B [27], curcumin-induced inhibition of NF-*κ*B could be responsible for the reduced levels of IL-17 observed in our experiments. Nevertheless, we previously reported that an increased expression of IDO occurs in human gastric mucosa colonized by *H. pylori* and that this fact is able to downregulate mucosal Th17 immune response [14]. Ciorba et al. observed that IDO inhibition in a mouse model of colitis worsens disease severity, suggesting that this enzyme plays a pivotal role in limiting inflammation in gastrointestinal tract [28]. Also, the expression of IDO gene is upregulated by interferon- (IFN-) *γ*, which is the predominant cytokine induced in human gastric mucosa during *H. pylori* infection and acts a pivotal role in the regulation of the Th1 response in the gastrointestinal tract [29]. Moreover, a role for IDO in controlling the immune responses and promoting a state of tolerance has been established [30, 31]. Based on this background, we decided to evaluate the expression of IDO in *ex vivo* cultured gastric biopsies challenged with curcumin. Data demonstrated that IDO expression was enhanced in *H. pylori*-infected treated samples compared with the untreated ones. Probably due to the chronic active inflammation sustained by *H. pylori* in human gastric mucosa and to the different experimental model, this result contrasts with that by Jeong et al., who showed that curcumin inhibits IDO in bone marrow-derived murine dendritic cells after stimulation by a single amount of IFN-*γ* [32]. Nevertheless, the documented anti-inflammatory effect of curcumin by a number of reports clearly supports our findings [33].

After noticing that curcumin affected IL-17 and IDO expression, we further investigated the relationship between the downregulation of IL-17 and the upregulation of IDO in *H. pylori*-infected samples treated with curcumin. We previously demonstrated that, in the same organ culture model of gastric biopsies from *H. pylori*-infected patients, treatment with the selective IDO inhibitor 1-MT was able to enhance the IL-17 production [14]. By adding 1-MT in the cultured biopsies from a subgroup of patients, treatment with curcumin was no longer capable to reduce IL-17 production. This result suggested that the downregulation of IL-17 levels exerted by curcumin during *H. pylori* infection is, at least in part, mediated by IDO expression. To the best of our knowledge, results from this study represent the first evidence which focuses on the interplay between curcumin and IDO in the human gastric mucosa. Although the precise mechanism by which curcumin increases the expression of IDO during *H. pylori* infection was not investigated in the present study, our findings allow for some speculations. Since no significant changes in IDO and IL-17 measurement were observed in biopsy specimens from uninfected patients, even treated with curcumin, it is strongly conceivable that there is an interaction between curcumin, IDO, and *H. pylori* in modulating the inflammatory pathway in the gastric mucosa. It is recognized that IDO limits Th1 responses towards external pathogens, thereby driving the host immune system towards a tolerogenic phenotype [34]. Specifically, the enhanced IDO expression in human gastric mucosa colonized by *H. pylori* has been shown to impair Th1/Th2 balance through both downregulating Th1 and Th17 and upregulating Th2 pathways, thus favouring the bacterial persistence [14]. Likewise, curcumin has been found to increase the presence of Treg cells, while inhibiting the secretion of Th1 and Th17 cytokines, in a mouse model of colitis [35]. More specifically, curcumin exerted therapeutic effects in inflamed colonic mucosa of mice by shifting the immune response from Th1 to Th2 [36]. Hence, an overlapping action profile between curcumin and IDO is noticeable, which may work synergistically to reduce inflammation sustained by a continuous proinflammatory stimuli such as that induced by *H. pylori*.

Although several *in vitro* and animal studies supported the anti-*H. pylori* activity of curcumin, it has been shown that curcumin failed to eradicate (or to improve the rate of eradication of) *H. pylori* in humans [37, 38]. Results of the present study could suggest some mechanisms responsible for this failure. Indeed, the novel finding that curcumin downregulates IL-17 by increasing IDO indicates that, while dampening the inflammatory burden, this synergistic mechanism promotes a tolerance phenotype helping bacterial evasion of the host surveillance mechanisms. Accordingly, Kao et al. suggested that an enhanced Treg induction in *H. pylori*-infected hosts causes an imbalance in the Th17/Treg axis, leading to suboptimal Th17 response which could be responsible for the failure of eradication [39]. Furthermore, an enhanced IDO expression has been found to impair *H. pylori*-induced Th1 response [14] as well as the *H. pylori*-induced expression of COX-2, which has also been showed to downregulate Th1 signalling pathway [40]. This is of particular interest, since Th1 immune response has been recently considered to be a major factor in eradicating *H. pylori* [41, 42]. Taken together, data of this study and those from the literature could explain the reason why the use of curcumin, in spite of its potential anti-*H. pylori* in *in vitro* and in animal studies, fails in the eradication of the bacterium in humans. The immune-mediated tolerance towards *H. pylori* prevails with the concomitant use of curcumin.

In conclusion, data of our study demonstrate that curcumin downregulates IL-17 levels by inducing the expression of IDO in the human gastric mucosa colonized by *H. pylori*. The abovementioned mechanism, while dampening the inflammatory burden, may facilitate the persistence of the bacterium.

## Data Availability

The data used to support the findings of this study are available from the corresponding author upon request.
